# Improved leukocyte classification in bone marrow cytology using convolutional neural network with contrast enhancement

**DOI:** 10.1038/s41598-025-12207-z

**Published:** 2025-08-19

**Authors:** Shahid Mehmood, Tariq Shahzad, Muhammad Zubair, Farman Matloob Khan, Muhammad Adnan Khan, Khmaies Ouahada, Amir H. Gandomi

**Affiliations:** 1 Department of Computer Science, Bahria University, Lahore, 54000 Pakistan; 2 Department of Computer Science, Riphah International University, Islamabad, Pakistan; 3 Department of Electrical and Electronic Engineering Science, University of Johannesburg, Auckland Park, P.O. Box 524, Johannesburg, 2006 South Africa; 4 College of Pharmacy, University of Sharjah, Sharjah, UAE; 5 College of Pharmacy, Universiti Teknologi MARA (UiTM), Shah Alam, Selangor Malaysia; 6 Pattern Recognition and Machine Learning Laboratory, Department of Software, Faculty of Artificial Intelligence and Software, Gachon University, Seongnam-si, 13557 Republic of Korea; 7 Faculty of Engineering & Information Technology, University of Technology Sydney, Sydney, NSW 2007 Australia; 8 University Research and Innovation Center (EKIK), Óbuda University, Budapest, 1034 Hungary; 9 Department of Computer Science, Khazar University, Baku, Azerbaijan

**Keywords:** Artificial intelligence (AI), Machine learning (ML), Deep learning (DL), Convolutional neural network (CNN), Transfer learning (TL), VGG16, Contrast limited adaptive histogram equalization (CLAHE), Leukocytes, Transfer learning (TL), Bone marrow, Cytology, Medical research, Computational science, Computer science

## Abstract

Leukocytes or white blood cells (WBCs) are the main components of the immune system that protect the human body from various infections caused by viruses, bacteria, fungi, and other microorganisms. There are five major types of leukocytes: basophils, lymphocytes, eosinophils, monocytes, and neutrophils. The precise identification and enumeration of each variety of WBCs are essential for the diagnosis and management of various conditions, including infectious diseases, immune disorders, immunological deficiencies, leukemia, and so forth. The conventional method of examining bone marrow cells by hematologists and pathologists using microscopy is tedious, time-consuming, and prone to variability among observers. Hence, there is a demand for a rapid and precise WBCs classification model. The proposed framework is highly accurate for the classification of leukocytes. A large dataset of leukocyte images was used in this study for training and testing. We used transfer learning to speed up the training process empowered with Contrast Limited Adaptive Histogram Equalization (CLAHE) technique to improve image quality and classification accuracy. The initial accuracy of the model was 81%. After the application of the CLAHE technique, the proposed approach significantly improved overall accuracy from 81 to 96.5% (15.5% improvement), outcompeting the state-of-the-art methods for leukocyte classification. Image contrast enhancement techniques, particularly CLAHE, improve the convolution neural network (CNN) model’s performance. The proposed model can significantly assist hematologists and pathologists in accurately identifying leukocytes, thereby aiding in the detection of blood disorders and enabling more effective treatment strategies.

## Introduction

The Cellular components of blood are classified into red blood cells (RBC), white blood cells (WBC), and platelets. Like other cellular components of blood, WBCs, also known as leukocytes, are produced in the bone marrow. The immune system relies heavily on leukocytes. These cells help the body fight infections and other diseases^1^. WBCs are classified into granulocytes and agranulocytes. Granulocytes include neutrophils, eosinophils, and basophils, while agranulocytes are lymphocytes and monocytes. Most WBCs comprise neutrophils, accounting for 50–70% of all WBCs. In peripheral blood, many neutrophils are in segmented (polymorphonuclear) form, while a small percentage appear as band cells^2^. These cells play a pivotal role in bacterial infections. An estimated 30–40% of the circulating WBCs are lymphocytes. Most of the lymphocytes are found in the lymphoid organs, such as the spleen and lymph nodes of the lymphatic system, in addition to their circulation in the blood. Subtypes of lymphocytes include B cells, T cells, and natural killer (NK) cells. Lymphocytes are the main players in the immune system by participating in innate immunity, humoral immunity, and cell-mediated immunity.

Furthermore, they also regulate several cells of the immune system. Additionally, between 2 and 10% of WBCs are monocytes, which develop into tissue macrophages. Monocytes and macrophages are involved in phagocytosis of invading bacteria or particles, removing virally infected or neoplastic cells, and producing several cytokines. Fewer than 5% of WBCs are eosinophils. Eosinophilia, i.e., increased count of eosinophils, is usually observed in allergic conditions, parasitic infestation, and some neoplastic diseases. Basophils, the least frequent form of circulating WBCs, comprise around 1–2% of WBCs. They have enormous histamine and serotonin-rich, purple-black granules in their cytoplasm. As a result, these cells play a part in allergic rhinitis, asthma, urticaria, and anaphylaxis, among other hypersensitivity reactions^3^.

The role of WBCs in the risk evaluation for critical coronary and vascular events, like stroke, makes them crucial for infectious, autoimmune, and oncologic illnesses as well. As a result, the study of WBCs is used to determine the diagnosis and recommend a course of treatment. The research also stresses how WBC population morphological dynamics may be used to identify changes from a healthy to a sick condition^4^. Therefore, while assessing WBC, it is crucial to consider the cells’ quantity, percentage, and shape. Leukopenia, characterized by a low WBC count, may manifest as a symptom or result from conditions such as bone marrow cancer, aplastic anemia, thyroid disorders, typhoid fever, and autoimmune diseases. WBC counts higher than normal, also called leukocytosis^5^, indicate a neoplasm or reaction. Leukocytosis has been linked to leukemia, polycystic ovarian disease, Addison’s disease, and bone marrow malformations^6^.

There are two ways to count WBCs: manually (using a hemocytometer) or automatically (using an analyzer). A standard blood test called a complete blood count^7^ is a common blood test that provides valuable insights into overall health by assessing various blood cell types. A standard WBC analyzer counts and identifies WBCs based on the size, cytoplasm, and nuclear structure of the cells. It is possible, however, for a size-based classification to be very misleading. The most reliable test for WBCs is peripheral smear morphology, which is still done manually. In addition to being time-consuming and subjective, this approach leaves room for human error. As a result, by utilizing automated recognition and diagnostic techniques to examine blood smear pictures, it is feasible to decrease the likelihood of human mistakes and the time required for the manual procedure^8^.

Deep convolutional neural networks (DCNNs) have recently achieved notable success in image recognition because of their robust natural structure and accessibility to a massive ImageNet dataset. There are 14 million images in the ImageNet dataset and 1,000 different item classes. Using raw pictures to train a brand-new deep CNN model is typically challenging. Pre-trained CNN models are useful in this situation because the learning process starts from patterns discovered by a big dataset from a comparable challenge instead of starting from the raw pictures^9^. Many researchers used pre-trained models to classify medical images, such as radiology and histopathology images, to detect and classify several diseases^10–16^. For instance, Liang et al.^17^, used CNNs in conjunction with recurrent neural networks (RNNs) to determine the type of WBCs by utilizing the long-term dependency connection between a few important aspects of images and their labels, not thoroughly investigated by traditional deep CNN approaches. Several pre-trained Classifiers were used in this investigation alongside transfer learning techniques. This model’s 90.70% classification performance compared to current techniques was poor compared to Classifiers like ResNet and GoogLeNet. Similarly, Livieris et al.^18^ evaluated the performance of semi-supervised learning (SSL) algorithms in blood cell classification. These algorithms apply a methodology of utilizing information from labeled data to explore hidden patterns of unlabeled data. The research results demonstrate that employing neighborhood strategies in a quasi-controlled learning strategy can improve performance. The study identified WBCs with 93.29% accuracy by implementing the SSL approach with a KNN classifier. The interclass commonalities in this technique make it less accurate in WBC-type classification.

An approach that relies on CNN hyper-parameter enhancement by the genetic algorithm was presented by Bani et al.^19^. This strategy picks up important characteristics that aid in differentiating the various WBC subtypesThe training accuracy of this method was 99.0%, whereas the testing accuracy stayed at 91%. This method’s flaws include its difficult implementation and poor testing accuracy. Banik et al.^20^ presented a mixed CNN model to identify different kinds of WBCs. As part of their model, three max-pooling layers were added to five deep convolutional layers, along with a hidden layer for each fully connected layer. Max-pooling was used to combine the feature maps of two convolutional layers to create an input for a fully connected layer. The accuracy of this model was 90.79%. Although it is quicker than the CNN-RNN model, the accuracy remains low due to similarities across classes and differences within classes.

Some researchers have utilized microscopic hyperspectral imaging (HSI) to classify WBCs by combining spectral and spatial features. Xueqi Hu et al.^21^ developed a method using morphological watershed segmentation to isolate nuclei and cytoplasm, then applied spectral characteristics with SVM for leukocyte classification. D. Yifan et al.^22^ enhanced this approach by integrating iterative data analysis and convex cone algorithms for improved segmentation and feature extraction, also using SVM for classification. While effective, these methods require complex preprocessing and segmentation to extract conventional features for accurate cell type recognition.

Many prior methods^23,24^ have used feature fusion to improve recognition accuracy, but few have implemented feature selection (FS), which reduces resource demands, misclassification rates, and workload^25–27^. However, some approaches have applied FS effectively for WBC identification. Shahin et al.^28^ combined CNN features with SVM classification, achieving 96.10% accuracy. FS methods like Mutual Information feature selection, ReliefF, Laplacian, and unsupervised discriminative feature selection (UDFS)^29–32^ further enhance feature-ranking. Ozyurt^33^ employed AlexNet^34^, VGG-16^35^, GoogleNet^36^, and ResNet^37^ with minimum redundancy maximum relevance (MRMR) for feature selection, achieving 95.29% accuracy using extreme learning machine (ELM)^38,39^, though this approach increased complexity by extracting deep features from fully connected (FC) layers only, missing structural WBC details.

Recent studies^40,41^ suggest using feature maps from higher convolutional layers, which contain semantically rich information, though less detail than lower layers. Jawahar et al.^42^ introduced ALNett, a model with depth-wise convolution and varying dilation rates, outperforming VGG16, ResNet-50, GoogleNet, and AlexNet in accuracy (91.13%), F1 score (0.96), and computational efficiency. This model’s structure includes convolution, max-pooling, and normalization stages, enabling efficient WBC feature extraction.

Matek et al.^43,44^ introduce a dataset comprising 171,374 microscopic images of bone marrow cells obtained from 945 patients with diverse hematological conditions. The researchers employ two CNN models, ResNeXt-50 and a sequential network, to categorize the bone marrow cell images into 21 morphological classes. The CNN models surpass the performance of a prior feature-based method and demonstrate high accuracy across most classes. Rigorous evaluation, considering only precise alignment between ground truth and network predictions, yields an average accuracy of 0.86 for the ResNeXt-50 model and 0.82 for the sequential model. These outcomes result from a fivefold cross-validation on the test dataset.

B. Ananthakrishnan^45^ introduced a Siamese network approach for categorizing bone marrow (BM) images into 21 distinct categories. Through extensive evaluation of the dataset comprising 170,000 images introduced by C. Matek^44^, their approach showcased impressive accuracy and generalization in predicting the assigned classes. In contrast to existing models such as ResNeXt-50 and XGG-Boost, which rely on feature extraction from individual images, Ananthakrishnan’s method emphasizes assessing image similarity and dissimilarity within and between classes. The Siamese neural network achieved a final accuracy of 91% during training and 84% during validation. However, the study acknowledges limitations, including class imbalance and notably low accuracy in a few classes.

In the work by S. Tripathi^46^, the challenge of limited automation in bone marrow cell categorization through deep learning techniques, typically confined to small datasets and specific disease classifications, is addressed. The authors employed the dataset introduced by C. Matek^44^ with 171,375 single-cell annotated images. The authors address these drawbacks by presenting a pipeline for classifying bone marrow cells. To balance class distribution, data augmentation techniques such as rotation, zooming, flipping, and translation were employed. The pipeline features the utilization of a CoAtNet model; this was contrasted with the EfficientNetV2 and ResNext50 baseline models. Although the model performed exceptionally well in the classification of some classes, it also presented poor accuracy for some other classes. Additionally, the CoAtNet model underwent analysis using explainability methods like SmoothGrad and Grad-CAM.

The following limitations have been noted in previous studies:

- Algorithms that are complex and inefficient^21,22,28,30,33^.
- Datasets with fewer classes or less diversity were utilized^17,23,27,29^.
- Several studies reported poor testing accuracy^17–20,24^.

Some of the main achievements of this paper can be stated as follows:

- Enhanced Classification Accuracy with CLAHE: We propose selective application of Contrast Limited Adaptive Histogram Equalization (CLAHE) as a pre-processing step for the enhancement of images to improve the learning capability of the pre-trained deep learning models. Conventional methods utilize contrast enhancement in all images indiscriminately, whereas our method employs CLAHE only if the testing accuracy is unsatisfactory which brings down the time and cost of computations without loss of accuracy. This selective deployment of CLAHE is a novel strategy that seeks to attain both high accuracy and high efficiency.
- Comprehensive WBC Classification across Six Classes: Most of the previous works concerning WBC classification are limited to four main classes of leukocytes most of the time not including basophils due to lack of data on them. Our work addresses this issue by performing the classification of six distinct types of WBCs, including basophils and two forms of neutrophils, namely band and segmented neutrophils in which the model becomes more relevant in practical clinical use. This comprehensive inclusion increases the applicability and extent of our model’s adoption.
- State-of-the-Art Performance on a Large Dataset: On a dataset comprising of 18,000 high-definition WBC images, we attained a classification accuracy of 96.5% which is higher than the previously reported models evaluated using the same dataset. This level of accuracy in clinical practice combined with the wide range of WBC classification categories represents a big step towards automated leukocyte differentiation.
- Efficient Use of Transfer Learning with VGG16: Instead of performing costly training on large sized medical image datasets, we present transfer learning employing the VGG16 architecture. This extends transfer learning beneficially in reducing computational requirements while enhancing model performance which reinforces the strategy of using VGG16 with CLAHE for WBC Classification.
- External Validation for Generalizability: We validated our framework on an external dataset in order to evaluate the robustness of the model, and achieved reasonable outcomes despite different imaging settings. Therefore, this external validation points to the generalizability and effectiveness of the model in different clinical imaging situations, which is an important aspect of application in the practice.

The remainder of this article is organized as follows: The methodology presents the details of the proposed model and the employed dataset, while the results and discussion section discusses the experimental environment and results. The last section concludes the paper.

## Methodology

### The model

**Fig. 1:**
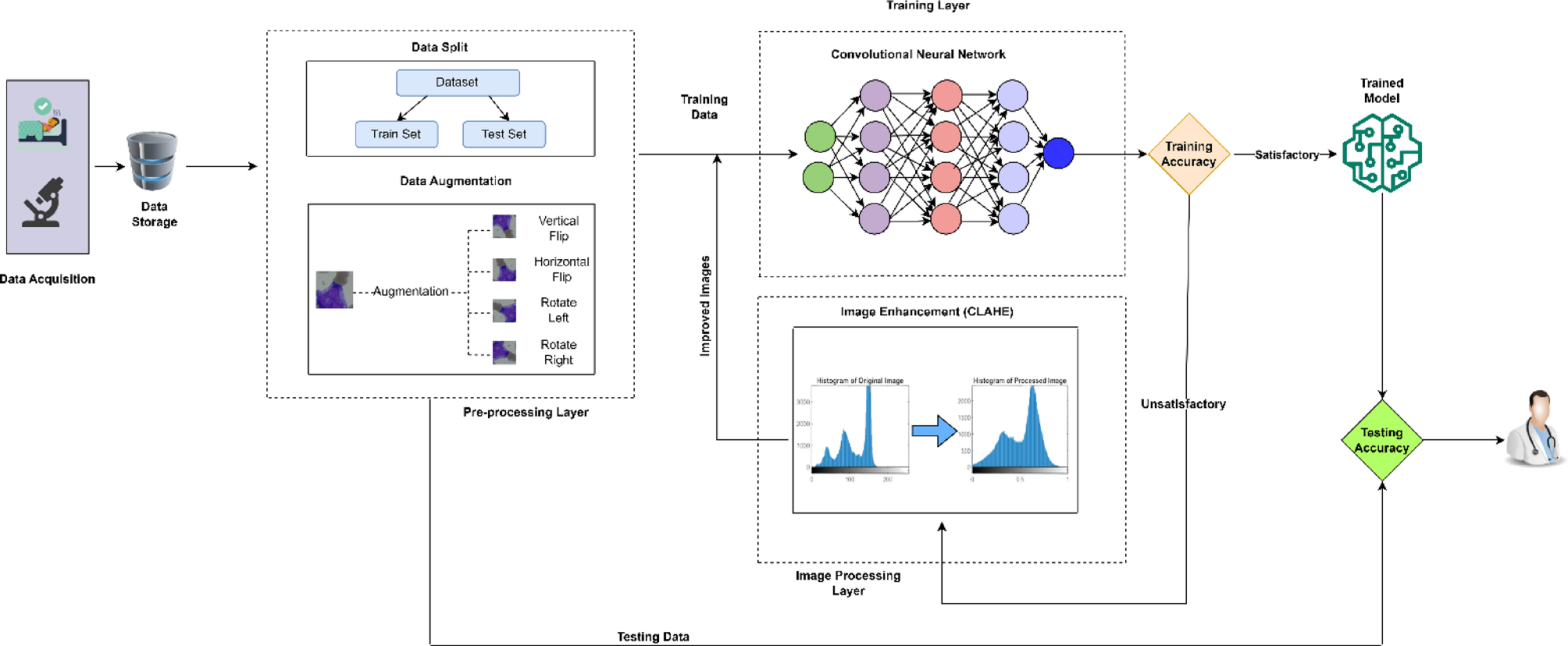
Proposed model for leukocyte classification.

Figure 1 presents the proposed model for leukocyte classification. The model is comprised of three layers. A description of each layer is presented below:

#### Data pre-processing layer

The first step in data pre-processing is to split the dataset into 80% images for training and 20% for testing. Secondly, the original dataset classes are highly imbalanced. To handle this imbalance, we performed down-sampling to get 3000 images from each class except the BAS class. On the other hand, the BAS class was up-sampled to 3000 images by using different augmentation schemes. We applied controlled variations in brightness and contrast. The brightness was randomly adjusted between 90% and 110% of the original brightness. The illumination was randomly modified by ± 10% to simulate variations without affecting key features.

#### Training layer

We employed the transfer learning technique to reduce training time and computational resources. We applied five pre-trained CNN models: AlexNet^34^, VGG16^35^, GoogleNet^36^, DenseNet^47^ and Inceptionresnet-V2^48^. These models have different architectures, but they share some common layers: input, convolutional, pooling, fully connected, and output. We briefly explain these layers below:

1. Input layer: This layer receives the input data for the CNN. It usually represents the image as a matrix of pixels in the neural network for image analysis.
2. Convolutional layer: The convolutional layer applies convolutional operations to the input data. In the convolution process, a filter slides over the input image and calculates the dot product at each position.

For a given position (j, k) in the input, the convolution operation is:

1 $$\:\left(h*y\right)\left(j,k\right)=\sum\:_{m}\:\sum\:_{n}\:h\left(m,n\right)\cdot\:y\left(j-m,k-n\right)$$

Here,

- (h) is the filter,
- (y) is the input image,
- (j, k) represents the position in the output feature map,
- (m) and (n) are the indices of the filter.

Activation functions in neural networks introduce non-linearities to the model. Data can then be analyzed for complex patterns and relationships. Some common activation functions are Sigmoid, Hyperbolic Tangent (tanh), and Rectified Linear Unit (ReLU).

3. Pooling layer: Pooling layers in neural networks, commonly followed by convolutional layers, reduce the spatial dimensions of the input data, lowering its computational load while retaining important features. In general, max and average pooling are the most common. Max pooling was primarily used in the proposed model.

Max pooling involves selecting the maximum value from a group of values within the input. If we have a window (pool size) of 2 × 2, the max pooling operation for a specific location (j, k) is given by:

2 $$\:\text{Max}\text{Pooling}\left(j,\text{k}\right)=max\left(y\left(j,\text{k}\right),y\left(j,\text{k}+1\right),y\left(\text{j}+1,\text{k}\right),y\left(\text{j}+1,\text{k}+1\right)\right)$$

Here, y is the input data, and the window slides over the input with a certain stride.

4. Fully connected layer: The data is integrated with this layer at the network’s outermost tiers to express classification clearly.
5. Output layer: In classification tasks, the output layer typically involves the use of a softmax activation function for multi-class classification. For each class *i*, the softmax function computes the probability *P*(class = *i*):

3 $$\:P(\text{c}\text{l}\text{a}\text{s}\text{s}=i)=\frac{{e}^{{z}_{i}}}{\sum\:_{j=1}^{C}\:{e}^{{z}_{j}}}$$

Here,

For class *i*, *z*_*i*_ is the logit (pre-activation).

There is a total of C classes.

In multi-class problems, softmax makes sure the predicted probabilities add up to 1. The predicted class is then often chosen as the one with the highest probability. In vector form, if z is the vector of logits, the softmax function is applied elementwise:

4 $$\:\text{S}\text{o}\text{f}\text{t}\text{m}\text{a}\text{x}\:{\left(z\right)}_{i}=\:\frac{{e}^{{z}_{i}}}{{\sum\:}_{j=1}^{C}{e}^{{z}_{j}}}$$

#### Image processing layer

This layer enhances the contrast of the dataset images and retrains the model using the improved images to increase classification accuracy. The image processing scheme applied for this purpose has been briefly described below:

#### Contrast limited adaptive histogram equalization

A contrast-limiting approach combined with AHE produces Contrast-Limited Adaptive Histogram Equalization (CLAHE)^49^. The primary purpose of CLAHE was to improve low-contrast medical images^50^. The contrast limiting of CLAHE sets it apart from regular AHE. The clip limit is a user-defined value that controls the amount of amplification in CLAHE. The histogram’s noise should be cleaned according to the clipping level, which also dictates the level of contrast to be increased. It’s really handy when there is a wide range of intensity levels in an image, and the details in the darker or brighter areas are hard to see. The CLAHE algorithm works by dividing the image into small, overlapping regions called tiles. For each tile, a histogram of pixel intensities is computed, and then the histogram is equalized. A contrast limit is applied during equalization to prevent noise amplification. This limit ensures that the contrast enhancement is applied in a controlled manner, preventing the creation of artifacts or unnatural-looking images. One of the advantages of CLAHE is its adaptability to different image characteristics. Since the algorithm operates on local regions instead of the entire image, it can handle images with varying lighting conditions or non-uniform illumination. This adaptability makes CLAHE suitable for a wide range of images, including those captured in challenging environments or under poor lighting conditions.

Suppose *I* (*x*,*y*) is the gray value of the pixel at (*x*,*y*) coordinates of the image. Let *Tij*​ represent the *i*-th row and the jth column tile in the image. The steps of CLAHE can be summarized as:

1. Divide the image into tiles: *I*_*Tij*_ (*x*,*y*) = *I* (*x*,*y*) for *x* ∈ [(*i* − 1) *M*, *iM* )] and *y* ∈ [(*j* − 1) *N*, *jN*)].
2. Calculate the histogram *H*_*Tij*_ ​​(*k*) for each tile *Tij*​, where *k* is the intensity value index.
3. Compute a clip limit, *C*, and then modify the histogram *H*_*Tij*_ ​​(*k*) by clipping and redistributing as necessary.
4. Perform histogram equalization on the modified histogram *H*_*Tij*_​​(*k*) to obtain the cumulative distribution function (CDF), *CDF*_*Tij*_​​ (*k*).
5. Apply the intensity transformation to each pixel in the tile:

5 $$\:P(\text{c}\text{l}\text{a}\text{s}\text{s}=i)=\frac{{e}^{{z}_{i}}}{\sum\:_{j=1}^{C}\:{e}^{{z}_{j}}}$$

Combine the enhanced tiles to form the final enhanced image. This can involve simple interpolation methods like bilinear interpolation to blend the transformed values between tiles.

##### CLAHE parameters used

- Clip Limit: 2.0, which controls the contrast enhancement to avoid excessive amplification of noise.
- Tile Grid Size: 8 × 8, dividing each image into smaller regions, allowing localized contrast adjustment.

**Fig. 2:**
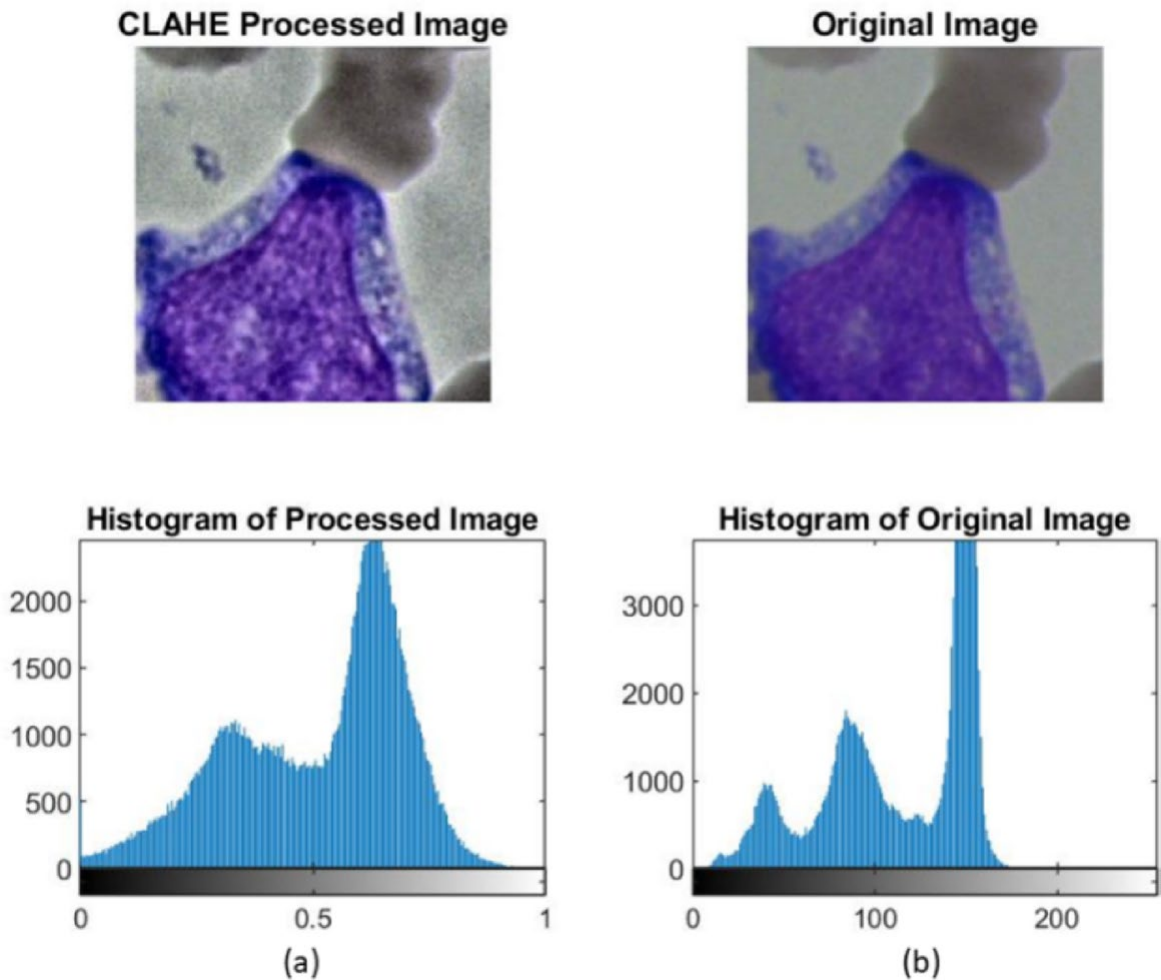
An example image along with its histogram pre-CLAHE and post-CLAHE.

A comparison of an original image with a processed image, along with their histograms, is shown in Fig. 2.

### Leukocyte images dataset

- The dataset used in the present study constitutes a subset of a larger dataset of 171,374 expert-annotated single-cell images. A total of 945 bone marrow cytological samples were taken from patients with a range of hematological disorders between 2011 and 2013 at MLL Munich Leukemia Laboratory^43^. Clinical data were used by the Declaration of Helsinki after written informed consent had been obtained from all patients. A single-cell image cannot be used to track a specific patient. An internal institutional review board at MLL Munich Leukemia Laboratory approved the study. With a median of 69.3 years and a mean of 65.6 years, the patients included in the study ranged in age from 18.1 to 92.2 years. Among the cohort, 59.8% of the patients were males and 40.1% were females, with 0.1% of them being of unknown gender^44^. The original dataset consists of 21 different classes of bone marrow cytomorphology images, six of which BAS (Basophil), EOS (Eosinophils), LYT (Lymphocyte), MON (Monocyte), NGB (Band neutrophil), NGS (Segmented neutrophil) contain WBCs. For this study, WBCs were classified using images from these six classes. The size of each image is 250 × 250 × 3. Table 1 provides the class names along with the corresponding number of images in each class.

**Table 1:** Dataset overview.

Cell type	Class title	Number of images
Basophil	BAS	3000
Eosinophil	EOS	3000
Lymphocyte	LYT	3000
Monocyte	MON	3000
Band neutrophil	NGB	3000
Segmented neutrophil	NGS	3000

## Results and discussion

Training and testing were conducted on a Windows laptop PC with an Intel Core i7-6500U CPU and 16 GB of DDR4 RAM. In this study, we used MATLAB R2020b to design and train the network, as well as to perform the experiments.

Our suggested technique generated encouraging outcomes compared to prior WBC categorization research. The classification performance of our model has been evaluated with the help of several performance metrics, including accuracy, precision, and recall. The subsequent equations describe the performance metrics employed^11^.

6 $$\:Precision=\:\frac{TP}{TP+FP}$$

7 $$\:Recall=\frac{TP}{TP+FN}$$

8 $$\:Accuracy=\frac{TP+TN}{TN+TP+FN+FP}$$

### Hyper parameters

The Adaptive moment estimation (Adam) optimization algorithm was used with a gradient decay factor of 0.9. We used a piecewise learn rate schedule with initial learn rate of 0.001, Learn rate drop factor of 0.5, learn rate drop period of 5. Each model was trained for 40 epochs with a minibatch size of 128.

### Results

We utilized five pre-trained models—AlexNet, VGG16, GoogleNet, DenseNet, and InceptionResNetV2. The results of these experiments, both pre and post the application of CLAHE, are detailed in Table 2. Prior to CLAHE, VGG16 led with the highest accuracy at 81%, while InceptionResNetv2 exhibited the lowest accuracy at 77.1%. AlexNet, DenseNet, GoogleNet, achieved an accuracy of 78.4%, 77.4%, and 77.8%, respectively. Post-CLAHE application, the models underwent evaluation again, and all showed improved accuracy. Notably, VGG16 stood out with a remarkable state-of-the-art accuracy of 95.8%. Hence, we discuss the result of the VGG16 model in further detail.

**Table 2:** Classification performance of the employed pre-trained CNN models.

Model	Pre-CLAHE accuracy	Post-CLAHE accuracy
Alexnet	78.4%	94.9%
Densenet	77.4%	95.4%
Googlenet	77.8%	95.5%
Inception-Resnet-V2	77.1%	95.3%
VGG16	81%	96.5%

**Fig. 3:**
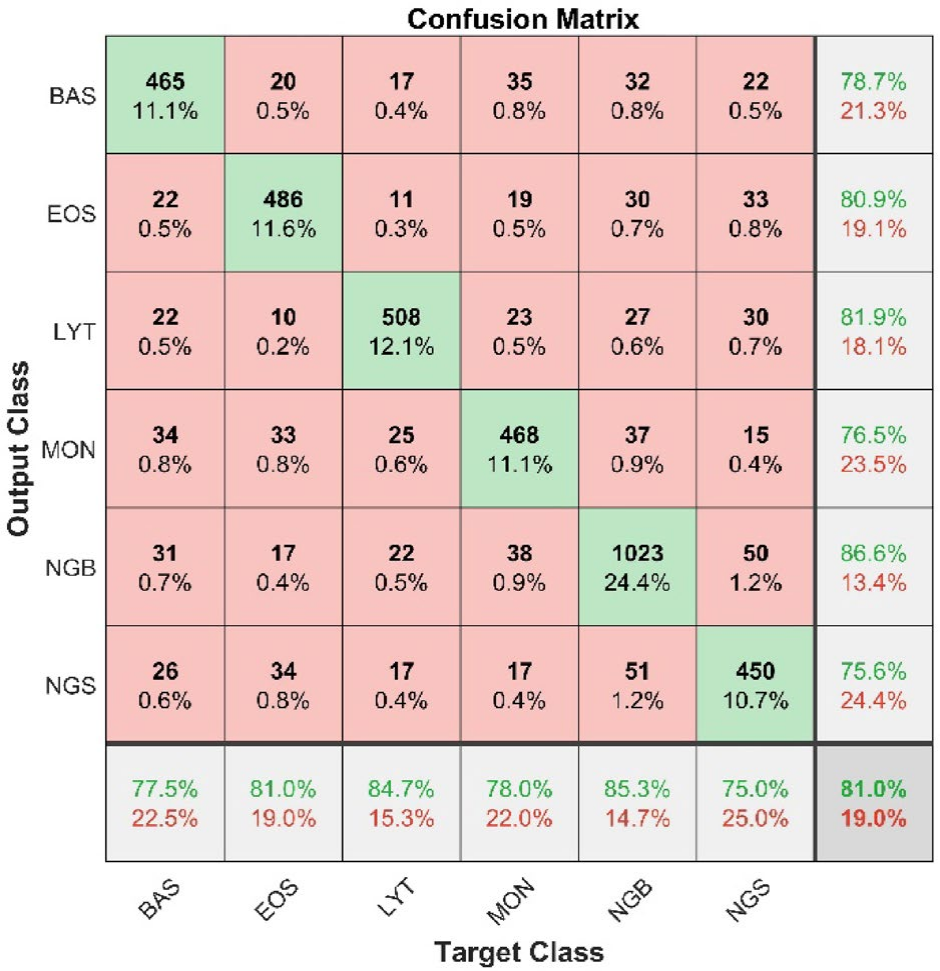
Model’s testing confusion matrix Pre-CLAHE implementation.

Figure 3 presents the confusion matrix, which shows an overall accuracy of 81%, along with each class’s recall and precision values. The rightmost column of the confusion matrix shows precision, while the bottom row presents recall values. The highest precision value is 86.6%, achieved by NGB class, followed by LYT (81.9%), EOS (80.9%), BAS (78.7%), MON (76.5%) and NGS (75.6%). At the same time, the highest recall value is attained by NGB (85.3%), followed by LYT (84.7%), EOS (81%), MON (78%), BAS (77.5%), and NGS (75%).

**Fig. 4:**
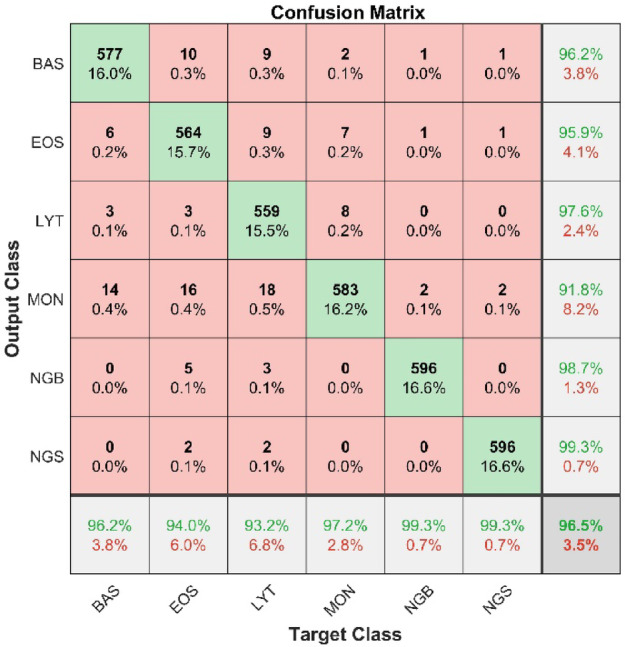
Model’s testing confusion matrix Post-CLAHE implementation.

CLAHE was applied to both training and testing images, and then the model was run on contrast-improved images for another 40 epochs. The results were significantly improved, and the overall accuracy of the model reached 96.5% (15.5% improvement), which is a state-of-the-art result for leukocyte classification. The model’s classification accuracy has been improved through the application of CLAHE. Figure 4 presents the post-CLAHE confusion matrix. In the confusion matrix, NGS shows the greatest improvement in precision (23.7%), followed by BAS (17.5%), EOS (15%), LYT (15.7%), MON (15.3%), and NGB (12.1). On the other hand, the maximum improvement in recall value is achieved by the NGS (24.3%), followed by MON (19.2%), BAS (18.7%), NGB (14%), EOS (13%), and LYT(8.5%).

### Disscussion

#### Computational complexity of cited algorithms

For each cited algorithm, we have reviewed available literature to extract or estimate the computational complexity (in terms of FLOPs or parameter count) where available. Table 3 presents a comparison of the most relevant algorithms. These complexity scores are approximations based on FLOPs or parameter counts, where available, and include the specific architectures and preprocessing techniques involved.

**Table 3:** Complexity score of the cited algorithms.

Algorithm	Model type	Complexity score (FLOPs)	Parameter count
Liang et al. (CNN-RNN)^17^	CNN + RNN	Approx. 10 GFLOPs (estimated)	~ 20 million
Bani et al. (Genetic-CNN)^19^	CNN with Genetic Search	Approx. 15 GFLOPs (estimated)	~ 30 million
Banik et al. (Mixed CNN)^20^	Mixed CNN	8–10 GFLOPs	~ 10 million
Hu et al. (HSI-SVM)^21^	HSI + SVM	Approx. 25 GFLOPs (due to HSI processing)	N/A (non-CNN)
Jawahar et al. (ALNett)^42^	Depth-wise CNN	Approx. 9 GFLOPs	~ 5 million

#### Computational complexity of the proposed algorithm

For our proposed model, which utilizes VGG16 with CLAHE-enhanced preprocessing, the approximate computational complexity is:

Complexity Score: 15.5 GFLOPs (VGG16 with CLAHE).

Parameter Count: ~138 million (VGG16 base model).

It is observed that the CLAHE step for preprocessing is helpful for the improvement of accuracy but comes with the cost of increased computing utilization. Nonetheless, in this study, the main aim of VGG16 was focused on leukocyte classification as it encompasses the greatest number of merits when it comes to feature extraction and classification accuracy which are critical for medical image analysis. Although, the VGG16 model has relatively high computational complexity (~ 15.5 GFLOPS) with a parameter count of about 138 million but there are various reasons why it is quite fit for this use despite the cost it demands.

Hierarchical Feature Extraction for Fine-Grained Classification: The major design component of the VGG16 networks is made of 3 × 3 convolutional layers in uniform sequential manner. This enables the model to build a layered structure to the features, from simple lines and textures at the initial layers, to more intricate shapes and structures further down the network. Such feature hierarchies are critical in classifying the different types of leukocytes, as they describe subtle morphological details that help in differentiating one type from the other.

Simplicity and Transferability of Architecture: The VGG16 architecture is simple and uniform since it consists of convolutional layers one after the other in stacks and simple max-pooling, which makes the feature extraction process very consistent and easy to understand. This makes it easier to use VGG16 for transfer learning. VGG16 was trained on large image datasets such as ImageNet which allows it to take advantage of the pre-trained weights. VGG16 performs particularly well with our leukocyte dataset as these features could be learned from VGG16’s pre-trained model without many labeled instances which is usually the hurdle with medical images. In addition, the simplicity also allows for the use of pre-processing methods such as CLAHE to improve the quality of the input images and increase the chances of retrieving more features which permits VGG16 to display a much better performance after enhancement.

Proven Performance in Medical Imaging Tasks: VGG16 has strong roots in medical imaging where correct morphological depiction plays a vital role. Its architecture has produced steady high-performance outcomes in the processing of complex and detailed images – particularly, image categorization of histopathological and radiological images. The relevance of the model to such applications speaks to its effectiveness and reliability for such tasks where other models like DenseNet consisting of dense connections or ResNet that has residual blocks may introduce complexities that are less effective in resolving finer details. VGG16 demonstrated a notable accuracy improvement from 81 to 96.5% with CLAHE-enhanced images in this study, underscoring its adaptability and high performance in a clinical imaging context. This accuracy is particularly important in medical applications where diagnostic precision can have a significant impact on patient outcomes.

Interpretability and Model Consistency: The simple layered structure of VGG16 brings in a level of interpretability that can be adopted in a clinical setting. In the case of GoogleNet and ResNet complicated architectures which have multiple paths and residuals, the output from VGG16 is less complex and straightforward making it easy to interpret and analyze it which improves transparency hence trust from the health care providers. This trait of the model is aligned with the recent developments in medical AI where explainable models are adopted because the decisions made are clinical in nature and thus sensitive. The performance of the model remains consistent when tested on other datasets and following different contrast enhancement processes which increases its trustworthiness.

Overcoming High Complexity through Selective Deployment: While VGG16 is computationally intensive, we have strategically deployed it in contexts where computational resources allow for high accuracy without real-time constraints. For instance, by applying CLAHE selectively and only when accuracy thresholds are unmet, we achieve a balance between computational demand and performance. This selective application mitigates some of the resource intensity typically associated with VGG16, making it practical for offline or batch processing environments commonly used in medical analysis.

Reasons for the Underperformance of Other Networks: Multi-branch structures, such as that of Inception-ResNet-v2, are incorporated in inception networks to obtain multi-scale features. This is useful in normal visual understanding problems, but it may obscure the exclusive attention on the texture and shape requirements for white blood cell classification. Further, the complexity of these networks may prove a hurdle in fine-tuning for the medical specific features resulting to the poor performance on our dataset. therefore, st with VGG16, AlexNet is a relatively shallow architecture therefore has limited ability to understand intricate details of images such as those of high resolution blood WBC. This factor of reduced depth has a negative impact on the ability of AlexNet to perform effective generalization for tasks geared towards fine medical classification. DenseNet is constructed upon the principle of dense interconnections of layers giving rise to duplicate feature maps. This configuration, although effective with the parameters, may not be the best suited for medical imaging which requires distinction of boundaries and textures very accurately. The same dense connectivity may also pose a problem in discriminating various features of classes where the morphologies are slightly different which is a critical aspect in classifying WBCs.

### Comparison of the proposed model with state-of-the-art

Several studies have investigated the role of deep learning models in the classification of leukocytes. Many studies employed CNN models for this classification task, while other research utilized alternative machine learning algorithms such as Support Vector Machines (SVM) and K-Nearest Neighbors (KNN). Despite this, our results are largely incomparable to those in the mentioned publications due to the fact that we used a dataset that was entirely different from those utilized in previous studies. However, Table 4 presents the comparative analysis of the classification results of three studies with the result of our proposed model. The results of these studies are comparable to our studies, as the same dataset^44^ has been used. Due to the CLAHE approach used in this study, the model has been able to function more effectively, which is the main reason for the increased performance. The proposed method consistently achieves remarkable precision and recall, with precision values ranging from 91.8 to 99.3% and recall values from 93.2 to 99.3%. Overall, the proposed method demonstrates consistent excellence in the classification of all six cell types and outperforms all other methods on the same dataset.

**Table 4:** Comparison of the proposed model with state-of-the-art literature.

	C. Matek et al.^43^		B. Ananthakrishnan et al.^45^		S. Tripathi et al.^46^		Proposed	
Class	Precision	Recall	Precision	Recall	Precision	Recall	Precision	Recall
Band neutrophils	91%	91%	85%	87%	97%	96%	98.7%	99.3%
Segmented neutrophils	95%	85%	97%	96%	95%	97%	99.3%	99.3%
Lymphocytes	90%	72%	62%	95%	94%	93%	97.6%	93.2
Monocytes	57%	70%	49%	61%	81%	79%	91.8%	97.2
Basophils	14%	64%	35%	34%	74%	70%	96.2%	96.2%
Eosinophils	85%	91%	93%	81%	91%	88%	95.9%	94%

## External validation

To examine the generalizability of our framework, it is imperative to analyze the system’s performance on a distinct dataset not employed during the training phase. At this time, scarcely any datasets are openly accessible that encompass individual BM cells in ample quantity with adequate imaging and annotation caliber, making it difficult to test the generalizability of our system. We tested our framework on an annotated dataset from Bodzas et al.^51^. From 36 peripheral blood smears with confirmed leukemia and 45 smears without leukemic pathology, a total of 12,986 microscopic blood smear images were obtained. These samples were from 78 research participants who had their identities removed. In this group, 18 patients were diagnosed with acute myeloid leukemia, 15 patients with acute lymphoid leukemia, and 45 patients with no clinical symptoms or a non-leukemic diagnosis. The raw images were stored in an uncompressed BMP file with a resolution of 5472 × 3648 pixels and a 24-bit color space. During the collecting stage, an experienced person manually annotated the dataset with the assistance of a domain specialist. Throughout the labeling phase, singular cell visuals were excerpted from each unprocessed visual, which diminished the ultimate dimensions of the visuals from 5472 × 3648 pixels to 1200 × 1200 pixels. We scaled all singular-cell visuals to 250 × 250 × 3 pixels and formulated predictions from the magnified visuals. The dataset is apportioned into nine categories, out of which six categories of leukocyte visuals were utilized for external validation.

**Fig. 5:**
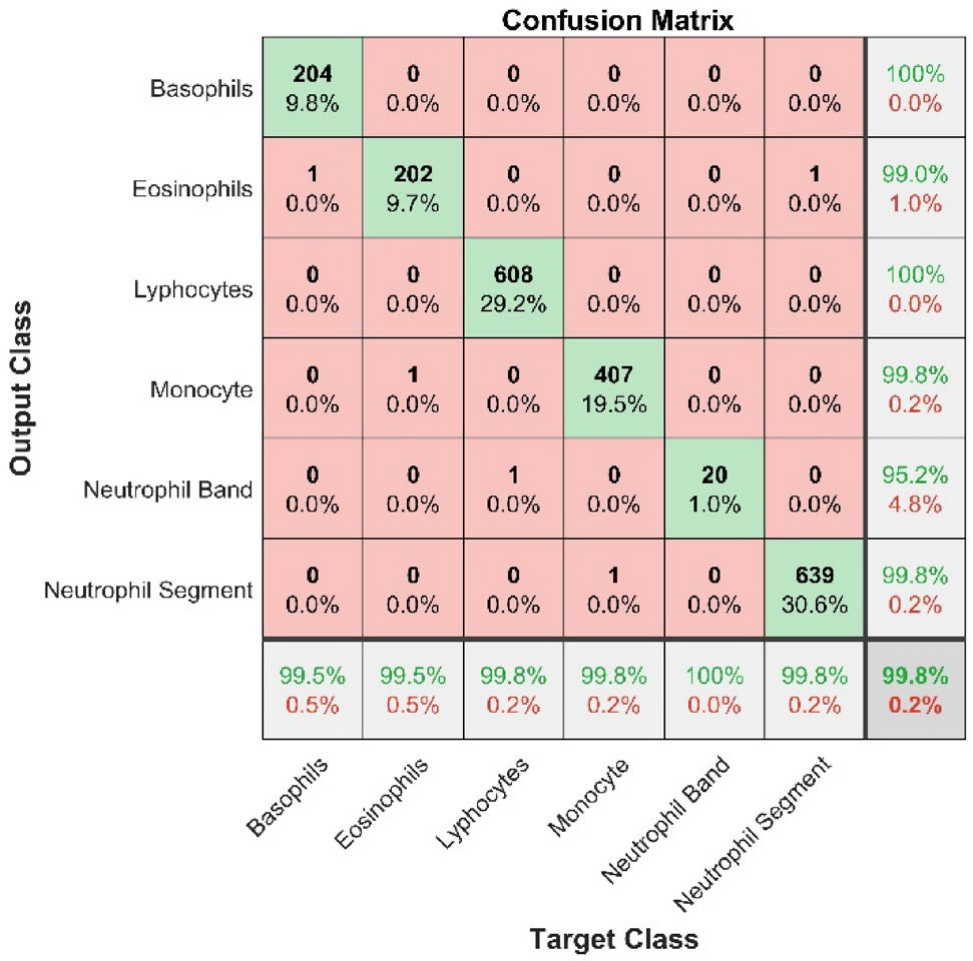
Model’s testing Confusion Matrix on external dataset.

Figure 5 shows the satisfactory performance of the classifier on the external dataset. Notably, the majority of cells are accurately sorted into their corresponding lineages. Considering the disparate imaging and annotation methodologies employed in the assembly of both datasets, an expected level of acceptable misclassification among individual lineage stages is anticipated. It is important to acknowledge that, in comparison to the internal dataset, the external evaluation dataset is relatively limited in size. Nonetheless, the classifier’s efficacy on the external dataset suggests that the model possesses the capability to generalize and identify instances where a definitive prediction cannot be established.

## Conclusion

This study sought to develop a highly accurate deep CNN model for identifying a variety of WBCs with potential applications in the diagnostic field. To achieve this goal, several pre-trained models, including AlexNet, GoogleNet, DenseNet, Inception-ResNet-v2, and VGG16, were employed. A total of 18,000 microscopic single-cell images of six different classes of leukocytes were used to train and evaluate the performance of these models. It was the VGG16 model that displayed the highest accuracy. Initially, the model exhibited a commendable accuracy of 81%. However, recognizing the importance of enhancing the quality of input images to boost the model’s performance further, CLAHE was applied. As a result, the model’s classification accuracy experienced a substantial leap, soaring to an impressive 96.5.8%, demonstrating a significant advancement over existing techniques. The outcome of this study shows a significant advancement over existing techniques on the same dataset. Despite the challenge posed by the limited available data, excellent performance on external data suggests that the method is generalizable and applicable to data obtained in different settings.

## Limitations and future work

This study is focused on the cell images obtained from a single laboratory prepared with the help of the same equipment.

Within that setting, our proposed method exhibited a very encouraging performance. It may be beneficial to incorporate larger and more diverse datasets to increase the potential use of this method in other laboratories and with various scanners. The performance and stability of the network, particularly for classes with limited samples in the employed dataset, may be improved through a multicentric study that includes a variety of scanner hardware. The primary objective of this study was to create and assess the proposed model. In the upcoming research, the computational complexity and resource demands of the model will be analyzed to determine its scale.

## Data Availability

The dataset & Simulation files used during the current study are available from the corresponding authors upon reasonable request.
